# Single-neuronal elements of speech production in humans

**DOI:** 10.1038/s41586-023-06982-w

**Published:** 2024-01-31

**Authors:** Arjun R. Khanna, William Muñoz, Young Joon Kim, Yoav Kfir, Angelique C. Paulk, Mohsen Jamali, Jing Cai, Martina L. Mustroph, Irene Caprara, Richard Hardstone, Mackenna Mejdell, Domokos Meszéna, Abigail Zuckerman, Jeffrey Schweitzer, Sydney Cash, Ziv M. Williams

**Affiliations:** 1 Department of Neurosurgery, Massachusetts General Hospital, Harvard Medical School, Boston, MA USA; 2 Harvard Medical School, Boston, MA USA; 3 Department of Neurology, Massachusetts General Hospital, Harvard Medical School, Boston, MA USA; 4 Harvard-MIT Division of Health Sciences and Technology, Boston, MA USA; 5 Harvard Medical School, Program in Neuroscience, Boston, MA USA

**Keywords:** Language, Extracellular recording

## Abstract

Humans are capable of generating extraordinarily diverse articulatory movement combinations to produce meaningful speech. This ability to orchestrate specific phonetic sequences, and their syllabification and inflection over subsecond timescales allows us to produce thousands of word sounds and is a core component of language^1,2^. The fundamental cellular units and constructs by which we plan and produce words during speech, however, remain largely unknown. Here, using acute ultrahigh-density Neuropixels recordings capable of sampling across the cortical column in humans, we discover neurons in the language-dominant prefrontal cortex that encoded detailed information about the phonetic arrangement and composition of planned words during the production of natural speech. These neurons represented the specific order and structure of articulatory events before utterance and reflected the segmentation of phonetic sequences into distinct syllables. They also accurately predicted the phonetic, syllabic and morphological components of upcoming words and showed a temporally ordered dynamic. Collectively, we show how these mixtures of cells are broadly organized along the cortical column and how their activity patterns transition from articulation planning to production. We also demonstrate how these cells reliably track the detailed composition of consonant and vowel sounds during perception and how they distinguish processes specifically related to speaking from those related to listening. Together, these findings reveal a remarkably structured organization and encoding cascade of phonetic representations by prefrontal neurons in humans and demonstrate a cellular process that can support the production of speech.

## Abstract (web summary)

Neuropixels recordings from the language-dominant prefrontal cortex reveal a structured organization of planned words, an encoding cascade of phonetic representations by prefrontal neurons in humans and a cellular process that could support the production of speech.

## Main

Humans can produce a remarkably wide array of word sounds to convey specific meanings. To produce fluent speech, linguistic analyses suggest a structured succession of processes involved in planning the arrangement and structure of phonemes in individual words^1,2^. These processes are thought to occur rapidly during natural speech and to recruit prefrontal regions in parts of the broader language network known to be involved in word planning^3–12^ and sentence construction^13–16^ and which widely connect with downstream areas that play a role in their motor production^17–19^. Cortical surface recordings have also demonstrated that phonetic features may be regionally organized^20^ and that they can be decoded from local-field activities across posterior prefrontal and premotor areas^21–23^, suggesting an underlying cortical structure. Understanding the basic cellular elements by which we plan and produce words during speech, however, has remained a significant challenge.

Although previous studies in animal models^24–26^ and more recent investigation in humans^27,28^ have offered an important understanding of how cells in primary motor areas relate to vocalization movements and the production of sound sequences such as song, they do not reveal the neuronal process by which humans construct individual words and by which we produce natural speech^29^. Further, although linguistic theory based on behavioural observations has suggested tightly coupled sublexical processes necessary for the coordination of articulators during word planning^30^, how specific phonetic sequences, their syllabification or inflection are precisely coded for by individual neurons remains undefined. Finally, whereas previous studies have revealed a large regional overlap in areas involved in articulation planning and production^31–35^, little is known about whether and how these linguistic process may be uniquely represented at a cellular scale^36^, what their cortical organization may be or how mechanisms specifically related to speech production and perception may differ.

Single-neuronal recordings have the potential to begin revealing some of the basic functional building blocks by which humans plan and produce words during speech and study these processes at spatiotemporal scales that have largely remained inaccessible^37–45^. Here, we used an opportunity to combine recently developed ultrahigh-density microelectrode arrays for acute intraoperative neuronal recordings, speech tracking and modelling approaches to begin addressing these questions.

## Neuronal recordings during natural speech

**Fig. 1:**
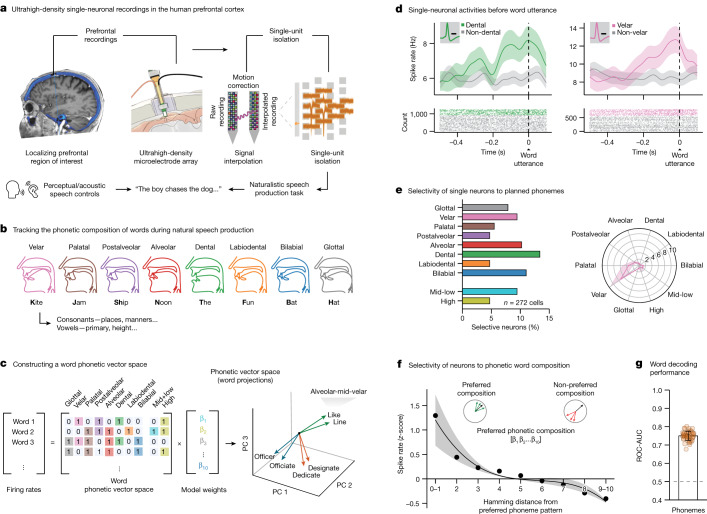
Tracking phonetic representations by prefrontal neurons during the production of natural speech. **a**, Left, single-neuronal recordings were confirmed to localize to the posterior middle frontal gyrus of language-dominant prefrontal cortex in a region known to be involved in word planning and production (Extended Data Fig. 1a,b); right, acute single-neuronal recordings were made using Neuropixels arrays (Extended Data Fig. 1c,d); bottom, speech production task and controls (Extended Data Fig. 2a). **b**, Example of phonetic groupings based on the planned places of articulation (Extended Data Table 1). **c**, A ten-dimensional feature space was constructed to provide a compositional representation of all phonemes per word. **d**, Peri-event time histograms were constructed by aligning the APs of each neuron to word onset at millisecond resolution. Data are presented as mean (line) values ± s.e.m. (shade). Inset, spike waveform morphology and scale bar (0.5 ms). **e**, Left, proportions of modulated neurons that selectively changed their activities to specific planned phonemes; right, tuning curve for a cell that was preferentially tuned to velar consonants. **f**, Average *z*-scored firing rates as a function of the Hamming distance between the preferred phonetic composition of the neuron (that producing largest change in activity) and all other phonetic combinations. Here, a Hamming distance of 0 indicates that the words had the same phonetic compositions, whereas a Hamming distance of 1 indicates that they differed by a single phoneme. Data are presented as mean (line) values ± s.e.m. (shade). **g**, Decoding performance for planned phonemes. The orange points provide the sampled distribution for the classifier’s ROC-AUC; *n* = 50 random test/train splits; *P* = 7.1 × 10^−18^, two-sided Mann–Whitney *U*-test. Data are presented as mean ± s.d. Source Data

**Extended Data Fig. 1:**
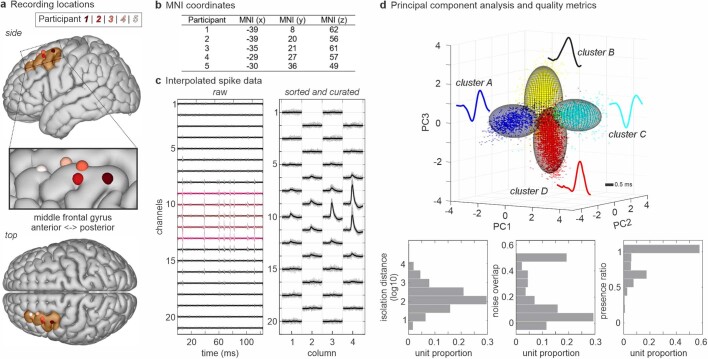
Single-unit isolations from the human prefrontal cortex using Neuropixels recordings. **a**. Individual recording sites on a standardized 3D brain model (FreeSurfer), on side (*top*), zoomed-in oblique (*inset*) and top (*bottom*) views. Recordings lay across the posterior middle frontal gyrus of the language-dominant prefrontal cortex and roughly ranged in distribution from alongside anterior area 55b to 8a. **b**. Recording coordinates for the five participants are given in MNI space. **c**. *Left*, representative example of raw, motion-corrected action potential traces recorded across neighbouring channels over time. *Right*, an example of overlayed spike waveform morphologies and their distribution across neighbouring channels recorded from a Neuropixels array. **d**. Isolation metrics for the recorded population (n = 272 units) together with an example of spikes from four concomitantly recorded units (labelled red, blue, cyan and yellow) in principal component space.

Single-neuronal recordings were obtained from the language-dominant (left) prefrontal cortex in participants undergoing planned intraoperative neurophysiology (Fig. 1a; section on ‘Acute intraoperative single-neuronal recordings’). These recordings were obtained from the posterior middle frontal gyrus^10,46–50^ in a region known to be broadly involved in word planning^3–12^ and sentence construction^13–16^ and to connect with neighbouring motor areas shown to play a role in articulation^17–19^ and lexical processing^51–53^ (Extended Data Fig. 1a). This region was traversed during recordings as part of planned neurosurgical care and roughly ranged in distribution from alongside anterior area 55b to 8a, with sites varying by approximately 10 mm (s.d.) across subjects (Extended Data Fig. 1b; section on ‘Anatomical localization of recordings’). Moreover, the participants undergoing recordings were awake and thus able to perform language-based tasks (section on ‘Study participants’), together providing an extraordinarily rare opportunity to study the action potential (AP) dynamics of neurons during the production of natural speech.

To obtain acute recordings from individual cortical neurons and to reliably track their AP activities across the cortical column, we used ultrahigh-density, fully integrated linear silicon Neuropixels arrays that allowed for high throughput recordings from single cortical units^54,55^. To further obtain stable recordings, we developed custom-made software that registered and motion-corrected the AP activity of each unit and kept track of their position across the cortical column (Fig. 1a, right)^56^. Only well-isolated single units, with low relative neighbour noise and stable waveform morphologies consistent with that of neocortical neurons were used (Extended Data Fig. 1c,d; section on ‘Acute intraoperative single-neuronal recordings’). Altogether, we obtained recordings from 272 putative neurons across five participants for an average of 54 ± 34 (s.d.) single units per participant (range 16–115 units).

**Extended Data Fig. 2:**
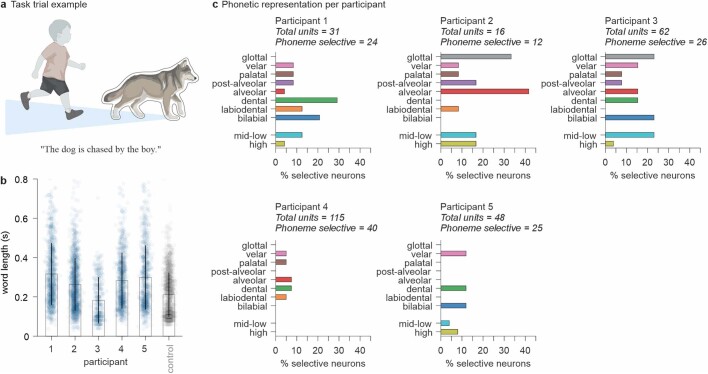
Naturalistic speech production task performance and phonetic selectivity across neurons and participants. **a**. A priming-based speech production task that provided participants with pictorial representations of naturalistic events and that had to be verbally described in specific order. The task trial example is given here for illustrative purposes (created with BioRender.com). **b**. Mean word production times across participants and their standard deviation of the mean. The blue bars and dots represent performances for the five participants in which recordings were acquired (n = 964, 1252, 406, 836, 805 words, respectively). The grey bar and dots represent healthy control (n = 1534 words). **c**. Percentage of modulated neurons that responded selectively to specific planned phonemes across participants. All participants possessed neurons that responded to various phonetic features (one-sided χ^2^ = 10.7, 6.9, 7.4, 0.5 and 1.3, p = 0.22, 0.44, 0.49, 0.97, 0.86, for participants 1–5, respectively).

Next, to study neuronal activities during the production of natural speech and to track their per word modulation, the participants performed a naturalistic speech production task that required them to articulate broadly varied words in a replicable manner (Extended Data Fig. 2a)^57^. Here, the task required the participants to produce words that varied in phonetic, syllabic and morphosyntactic content and to provide them in a structured and reproducible format. It also required them to articulate the words independently of explicit phonetic cues (for example, from simply hearing and then repeating the same words) and to construct them de novo during natural speech. Extra controls were further used to evaluate for preceding word-related responses, sensory–perceptual effects and phonetic–acoustic properties as well as to evaluate the robustness and generalizability of neuronal activities (section on ‘Speech production task’).

Together, the participants produced 4,263 words for an average of 852.6 ± 273.5 (s.d.) words per participant (range 406–1,252 words). The words were transcribed using a semi-automated platform and aligned to AP activity at millisecond resolution (section on ‘Audio recordings and task synchronization’)^51^. All participants were English speakers and showed comparable word-production performances (Extended Data Fig. 2b).

## Representations of phonemes by neurons

To first examine the relation between single-neuronal activities and the specific speech organs involved^58,59^, we focused our initial analyses on the primary places of articulation^60^. The places of articulation describe the points where constrictions are made between an active and a passive articulator and are what largely give consonants their distinctive sounds. Thus, for example, whereas bilabial consonants (/p/ and /b/) involve the obstruction of airflow at the lips, velar consonants are articulated with the dorsum of the tongue placed against the soft palate (/k/ and /g/; Fig. 1b). To further examine sounds produced without constriction, we also focused our initial analyses on vowels in relation to the relative height of the tongue (mid-low and high vowels). More phonetic groupings based on the manners of articulation (configuration and interaction of articulators) and primary cardinal vowels (combined positions of the tongue and lips) are described in Extended Data Table 1.

Next, to provide a compositional phonetic representation of each word, we constructed a feature space on the basis of the constituent phonemes of each word (Fig. 1c, left). For instance, the words ‘like’ and ‘bike’ would be represented uniquely in vector space because they differ by a single phoneme (‘like’ contains alveolar /l/ whereas ‘bike’ contains bilabial /b/; Fig. 1c, right). The presence of a particular phoneme was therefore represented by a unitary value for its respective vector component, together yielding a vectoral representation of the constituent phonemes of each word (section on ‘Constructing a word feature space’). Generalized linear models (GLMs) were then used to quantify the degree to which variations in neuronal activity during planning could be explained by individual phonemes across all possible combinations of phonemes per word (section on ‘Single-neuronal analysis’).

**Extended Data Fig. 3:**
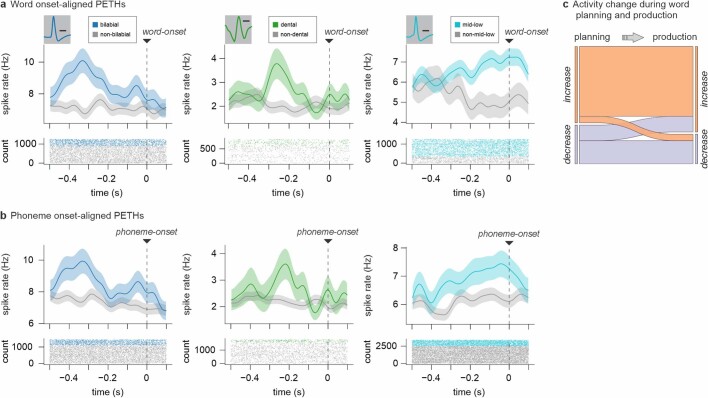
Examples of single-neuronal activities and their temporal dynamics. **a**. Peri-event time histograms were constructed by aligning the action potentials of each neuron to word onset. Data are presented as mean (line) values ± standard error of the mean (shade). Examples of three representative neurons that selectively changed their activity to specific planned phonemes. *Inset*, spike waveform morphology and scale bar (0.5 ms). **b**. Peri-event time histogram and action potential raster for the same neurons above but now aligned to the onset of the articulated phonemes themselves. Data are presented as mean (line) values ± standard error of the mean (shade). **c**. Sankey diagram displaying the proportions of neurons (n = 56) that displayed a change in activity polarity (increases in orange and decreases in purple) from planning to production.

**Extended Data Fig. 4:**
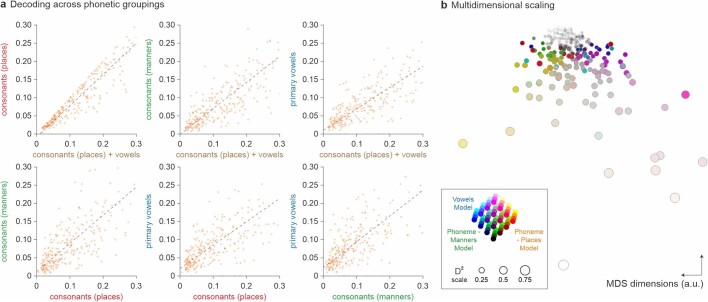
Generalizability of explanatory power across phonetic groupings for consonants and vowels. **a**. Scatter plots of the model explanatory power (D^2^) for different phonetic groupings across the cell population (n = 272 units). Phonetic groupings were based on the planned (i) places of articulation of consonants and/or vowels (ii) manners of articulation of consonants and (iii) primary cardinal vowels (Extended Data Table 1). Model D^2^ explanatory power across all phonetic groupings were significantly correlated (from top left to bottom right, p = 1.6×10^−146^, p = 2.8×10^−70^, p = 6.1×10^−54^, p = 1.4×10^−57^, p = 2.3×10^−43^ and p = 5.9×10^−43^, two-sided tests of Spearman rank-order correlations). Spearman’s ρ are 0.96, 0.83, 0.77, respectively for left to right top panels and 0.78, 0.71, 0.71, respectively for left to right bottom panels (dashed regression lines). Among phoneme-selective neurons, the planned places of articulation provided the highest explanatory power (two-sided Wilcoxon signed-rank test of model D^2^ values, W = 716, p = 7.9×10^−16^) and the best model fits (two-sided Wilcoxon signed-rank test of AIC, W = 2255, p = 1.3×10^−5^) compared to manners of articulation. They also provided the highest explanatory power (two-sided Wilcoxon signed-rank test of model D^2^ values, W = 846, p = 9.7×10^−15^) and fits (two-sided Wilcoxon signed-rank test of AIC, W = 2088, p = 2.0×10^−6^) compared to vowels. **b**. Multidimensional scaling (MDS) representation of all neurons across phonetic groupings. Neurons with similar response characteristics are plotted closer together. The hue of each point reflects the degree of selectivity to specific phonetic features. Here, the colour scale for places of articulation is provided in red, manners of articulation in green and vowels in blue. The size of each point reflects the magnitude of the maximum explanatory power in relation to each cell’s phonetic selectivity (maximum D^2^ for places of articulation of consonants and/or vowels, manners of articulation of consonants and primary cardinal vowels).

**Extended Data Fig. 5:**
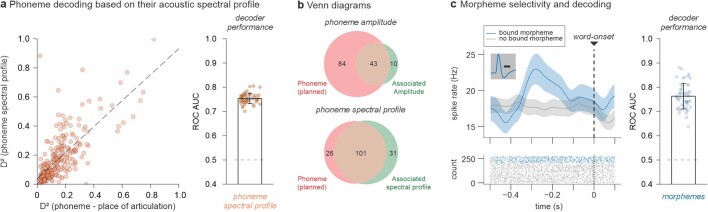
Explanatory power for the acoustic–phonetic properties of phonemes and neuronal tuning to morphemes. **a**. *Left*, scatter plot of the D^2^ explanatory power of neurons for planned phonemes and their observed spectral frequencies during articulation (n = 272 units; Spearman’s ρ = 0.75, p = 9.3×10^−50^, two-sided test of Spearman rank-order correlation). *Right*, decoding performances for the spectral frequency of phonemes (n = 50 random test/train splits; p = 7.1×10^−18^, two-sided Mann–Whitney U-test). Data are presented as mean values ± standard error of the mean. **b**. Venn diagrams of neurons that were modulated by phonemes during planning and those that were modulated by the spectral frequency (left) and amplitude (right) of the phonemes during articulation. **c**. *Left*, peri-event time histogram and raster for a representative neuron exhibiting selectivity to words that contained bound morphemes (for example, *–ing*, *–ed*) compared to words that did not. Data are presented as mean (line) values ± standard error of the mean (shade). *Inset*, spike waveform morphology and scale bar (0.5 ms). *Right*, decoding performance distribution for morphemes (n = 50 random test/train splits; p = 1.0×10^−17^, two-sided Mann–Whitney U-test). Data are presented as mean values ± standard deviation.

Overall, we find that the firing activities of many of the neurons (46.7%, *n* = 127 of 272 units) were explained by the constituent phonemes of the word before utterance (−500 to 0 ms); GLM likelihood ratio test, *P* < 0.01); meaning that their activity patterns were informative of the phonetic content of the word. Among these, the activities of 56 neurons (20.6% of the 272 units recorded) were further selectively tuned to the planned production of specific phonemes (two-sided Wald test for each GLM coefficient, *P* < 0.01, Bonferroni-corrected across all phoneme categories; Fig. 1d,e and Extended Data Figs. 2 and 3). Thus, for example, whereas certain neurons changed their firing rate when the upcoming words contained bilabial consonants (for example, /p/ or /b/), others changed their firing rate when they contained velar consonants. Of these neurons, most encoded information both about the planned places and manners of articulation (*n* = 37 or 66% overlap, two-sided hypergeometric test, *P* < 0.0001) or planned places of articulation and vowels (*n* = 27 or 48% overlap, two-sided hypergeometric test, *P* < 0.0001; Extended Data Fig. 4). Most also reflected the spectral properties of the articulated words on a phoneme-by-phoneme basis (64%, *n* = 36 of 56; two-sided hypergeometric test, *P* = 1.1 × 10^−10^; Extended Data Fig. 5a,b); together providing detailed information about the upcoming phonemes before utterance.

Because we had a complete representation of the upcoming phonemes for each word, we could also quantify the degree to which neuronal activities reflected their specific combinations. For example, we could ask whether the activities of certain neurons not only reflected planned words with velar consonants but also words that contained the specific combination of both velar and labial consonants. By aligning the activity of each neuron to its preferred phonetic composition (that is, the specific combination of phonemes to which the neuron most strongly responded) and by calculating the Hamming distance between this and all other possible phonetic compositions across words (Fig. 1c, right; section on ‘Single-neuronal analysis’), we find that the relation between the vectoral distances across words and neuronal activity was significant (two-sided Spearman’s *ρ* = −0.97, *P* = 5.14 × 10^−7^; Fig. 1f). These neurons therefore seemed not only to encode specific planned phonemes but also their specific composition with upcoming words.

Finally, we asked whether the constituent phonemes of the word could be robustly decoded from the activity patterns of the neuronal population. Using multilabel decoders to classify the upcoming phonemes of words not used for model training (section on ‘Population modelling’), we find that the composition of phonemes could be predicted from neuronal activity with significant accuracy (receiver operating characteristic area under the curve; ROC-AUC = 0.75 ± 0.03 mean ± s.d. observed versus 0.48 ± 0.02 chance, *P* < 0.001, two-sided Mann–Whitney *U*-test; Fig. 1g). Similar findings were also made when examining the planned manners of articulation (AUC = 0.77 ± 0.03, *P* < 0.001, two-sided Mann–Whitney *U*-test), primary cardinal vowels (AUC = 0.79 ± 0.04, *P* < 0.001, two-sided Mann–Whitney *U*-test) and their spectral properties (AUC = 0.75 ± 0.03, *P* < 0.001, two-sided Mann–Whitney *U*-test; Extended Data Fig. 5a, right). Taken together, these neurons therefore seemed to reliably predict the phonetic composition of the upcoming words before utterance.

## Motoric and perceptual processes

**Extended Data Fig. 6:**
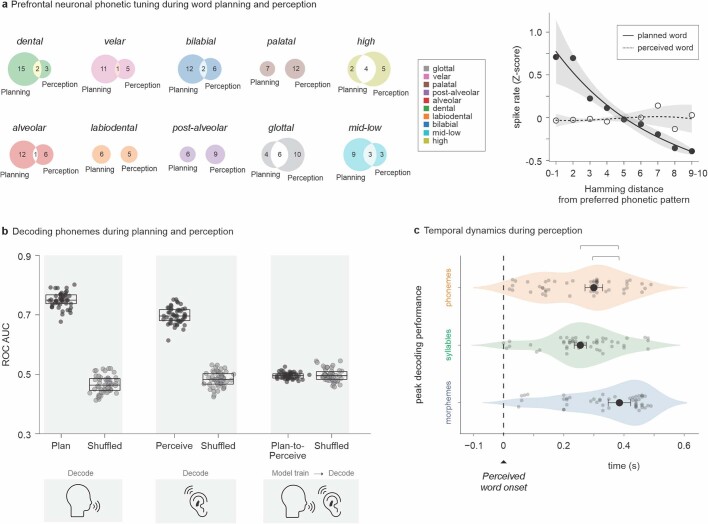
Phonetic representations of words during speech perception and the comparison of speaking to listening. **a**. *Left*, Venn diagrams of neurons that selectively changed their activity to specific phonemes during word planning (−500:0 ms from word utterance onset) and perception (0:500 ms from word utterance onset). *Right*, average z-scored firing rate for selective neurons during word planning (black) and perception (grey) as a function of the Hamming distance. Here, the Hamming distance was based on the neurons’ preferred phonetic compositions during production and compared for the same neurons during perception. Data are presented as mean (line) values ± standard error of the mean (shade). **b**. *Left*, classifier decoding performances for selective neurons during word planning. The points provide the sampled distribution for the classifier’s ROC-AUC values (black) compared to random chance (grey; n = 50 random test/train splits; p = 7.1×10^−18^, two-sided Mann–Whitney U-test). *Middle*, decoding performance for selective neurons during perception (n = 50 random test/train splits; 7.1×10^−18^, two-sided Mann–Whitney U-test). *Right*, word planning-perception model-switch decoding performances for selective neurons. Here, models were trained on neural data for specific phonemes during planning and then used to decode those same phonemes during perception (n = 50 random test/train splits; p > 0.05, two-sided Mann–Whitney U-test; **Methods**). The boundaries and midline of the boxplots represent the 25^th^ and 75^th^ percentiles and the median, respectively. **c**. Peak decoding performance for phonemes, syllables and morphemes as a function of time from perceived word onset. Peak decoding for morphemes was observed significantly later than for phonemes and syllables during perception (n = 50 random test/train splits; two-sided Kruskal–Wallis, H = 14.8, p = 0.00062). Data are presented here as median (dot) values ± bootstrapped standard error of the median.

Neurons that reflected the phonetic composition of the words during planning were largely distinct from those that reflected their composition during perception. It is possible, for instance, that similar response patterns could have been observed when simply hearing the words. Therefore, to test for this, we performed an extra ‘perception’ control in three of the participants whereby they listened to, rather than produced, the words (*n* = 126 recorded units; section on ‘Speech production task’). Here, we find that 29.3% (*n* = 37) of the neurons showed phonetic selectively during listening (Extended Data Fig. 6a) and that their activities could be used to accurately predict the phonemes being heard (AUC = 0.70 ± 0.03 observed versus 0.48 ± 0.02 chance, *P* < 0.001, two-sided Mann–Whitney *U*-test; Extended Data Fig. 6b). We also find, however, that these cells were largely distinct from those that showed phonetic selectivity during planning (*n* = 10; 7.9% overlap) and that their activities were uninformative of phonemic content of the words being planned (AUC = 0.48 ± 0.01, *P* = 0.99, two-sided Mann–Whitney *U*-test; Extended Data Fig. 6b). Similar findings were also made when replaying the participant’s own voices to them (‘playback’ control; 0% overlap in neurons); together suggesting that speaking and listening engaged largely distinct but complementary sets of cells in the neural population.

Given the above observations, we also examined whether the activities of the neurons could have been explained by the acoustic–phonetic properties of the preceding spoken words. For example, it is possible that the activities of the neuron may have partly reflected the phonetic composition of the previous articulated word or their motoric components. Thus, to test for this, we repeated our analyses but now excluded words in which the preceding articulated word contained the phoneme being decoded (section on ‘Single-neuronal analysis’) and find that decoding performance remained significant (AUC = 0.72 ± 0.1, *P* < 0.001, two-sided Mann–Whitney *U*-test). We also find that decoding performance remained significant when constricting (−400 to 0 ms window instead of −500:0 ms; AUC = 0.72 ± 0.1, *P* < 0.001, two-sided Mann–Whitney *U*-test) or shifting the analysis window closer to utterance (−300 to +200 ms window results in AUC = 0.76 ± 0.1, *P* < 0.001, two-sided Mann–Whitney *U*-test); indicating that these neurons coded for the phonetic composition of the upcoming words.

## Syllabic and morphological features

**Fig. 2:**
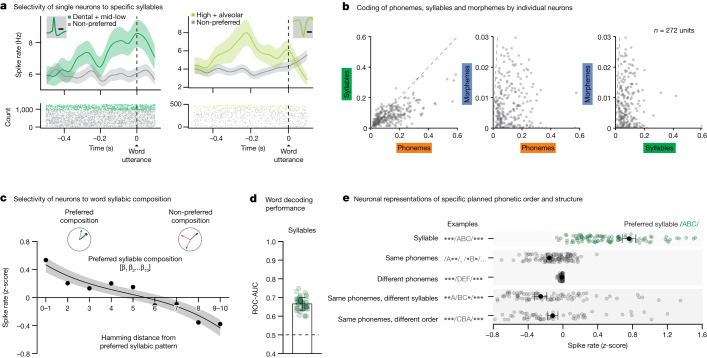
Cells that encode the arrangement and segmentation of phonemes into distinct syllables. **a**, Peri-event time histograms were constructed by aligning the APs of each neuron to word onset. Data are presented as mean (line) values ± s.e.m. (shade). Examples of two representative neurons which selectively changed their activity to specific planned syllables. Inset, spike waveform morphology and scale bar (0.5 ms). **b**, Scatter plots of *D*^2^ values (the degree to which specific features explained neuronal response, *n* = 272 units) in relation to planned phonemes, syllables and morphemes. **c**, Average *z*-scored firing rates as a function of the Hamming distance between the preferred syllabic composition and all other compositions of the neuron. Data are presented as mean (line) values ± s.e.m. (shade). **d**, Decoding performance for planned syllables. The orange points provide the sampled distribution for the classifier’s ROC-AUC values (*n* = 50 random test/train splits; *P* = 7.1 × 10^−18^ two-sided Mann–Whitney *U*-test). Data are presented as mean ± s.d. **e**, To evaluate the selectivity of neurons to specific syllables, their activities were further compared for words that contained the preferred syllable of each neuron (that is, the syllable to which they responded most strongly; green) to (i) words that contained one or more of same individual phonemes but not necessarily their preferred syllable, (ii) words that contained different phonemes and syllables, (iii) words that contained the same phonemes but divided across different syllables and (iv) words that contained the same phonemes in a syllable but in different order (grey). Neuronal activities across all comparisons (to green points) were significant (*n* = 113; *P* = 6.2 × 10^−20^, 8.8 × 10^−20^, 4.2 × 10^−20^ and 1.4 × 10^−20^, for the comparisons above, respectively; two-sided Wilcoxon signed-rank test). Data are presented as mean (dot) values ± s.e.m. Source Data

To transform sets of consonants and vowels into words, the planned phonemes must also be arranged and segmented into distinct syllables^61^. For example, even though the words ‘casting’ and ‘stacking’ possess the same constituent phonemes, they are distinguished by their specific syllabic structure and order. Therefore, to examine whether neurons in the population may further reflect these sublexical features, we created an extra vector space based on the specific order and segmentation of phonemes (section on ‘Constructing a word feature space’). Here, focusing on the most common syllables to allow for tractable neuronal analysis (Extended Data Table 1), we find that the activities of 25.0% (*n* = 68 of 272) of the neurons reflected the presence of specific planned syllables (two-sided Wald test for each GLM coefficient, *P* < 0.01, Bonferroni-corrected across all syllable categories; Fig. 2a,b). Thus, whereas certain neurons may respond selectively to a velar-low-alveolar syllable, other neurons may respond selectively to an alveolar-low-velar syllable. Together, the neurons responded preferentially to specific syllables when tested across words (two-sided Spearman’s *ρ* = −0.96, *P* = 1.85 × 10^−6^; Fig. 2c) and accurately predicted their content (AUC = 0.67 ± 0.03 observed versus 0.50 ± 0.02 chance, *P* < 0.001, two-sided Mann–Whitney *U*-test; Fig. 2d); suggesting that these subsets of neurons encoded information about the syllables.

Next, to confirm that these neurons were selectively tuned to specific syllables, we compared their activities for words that contained the preferred syllable of each neuron (for example, /d-iy/) to words that simply contained their constituent phonemes (for example, d or iy). Thus, for example, if these neurons reflected individual phonemes irrespective of their specific order, then we would observe no difference in response. On the basis of these comparisons, however, we find that the responses of the neurons to their preferred syllables was significantly greater than to that of their individual constituent phonemes (*z*-score difference 0.92 ± 0.04; two-sided Wilcoxon signed-rank test, *P* < 0.0001; Fig. 2e). We also tested words containing syllables with the same constituent phonemes but in which the phonemes were simply in a different order (for example, /g-ah-d/ versus /d-ah-g/) but again find that the neurons were preferentially tuned to specific syllables (*z*-score difference 0.99 ± 0.06; two-sided Wilcoxon signed-rank test, *P* < 1.0 × 10^−6^; Fig. 2e). Then, we examined words that contained the same arrangements of phonemes but in which the phonemes themselves belonged to different syllables (for example, /r-oh-b/ versus r-oh/b-; accounting prosodic emphasis) and similarly find that the neurons were preferentially tuned to specific syllables (*z*-score difference 1.01 ± 0.06; two-sided Wilcoxon signed-rank test, *P* < 0.0001; Fig. 2e). Therefore, rather than simply reflecting the phonetic composition of the upcoming words, these subsets of neurons encoded their specific segmentation and order in individual syllables.

Finally, we asked whether certain neurons may code for the inclusion of morphemes. Unlike phonemes, bound morphemes such as ‘–ed’ in ‘directed’ or ‘re–’ in ‘retry’ are capable of carrying specific meanings and are thus thought to be subserved by distinct neural mechanisms^62,63^. Therefore, to test for this, we also parsed each word on the basis of whether it contained a suffix or prefix (controlling for word length) and find that the activities of 11.4% (*n* = 31 of 272) of the neurons selectively changed for words that contained morphemes compared to those that did not (two-sided Wald test for each GLM coefficient, *P* < 0.01, Bonferroni-corrected across morpheme categories; Extended Data Fig. 5c). Moreover, neural activity across the population could be used to reliably predict the inclusion of morphemes before utterance (AUC = 0.76 ± 0.05 observed versus 0.52 ± 0.01 for shuffled data, *P* < 0.001, two-sided Mann–Whitney *U*-test; Extended Data Fig. 5c), together suggesting that the neurons coded for this sublexical feature.

## Spatial distribution of neurons

**Extended Data Fig. 7:**
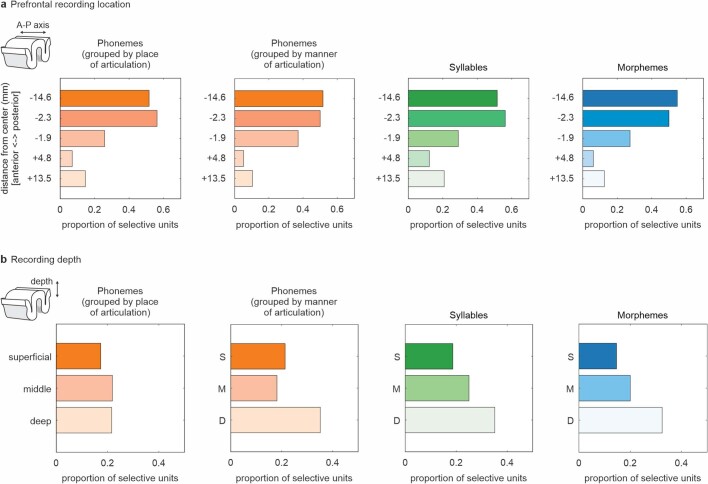
Spatial distribution of representations based on cortical location and depth. **a**. Relationship between recording location along the rostral–caudal axis of the prefrontal cortex and the proportion of neurons that displayed selectivity to specific phonemes, syllables and morphemes. Neurons that displayed selectivity were more likely to be found posteriorly (one-sided χ^2^ test, p = 2.6×10^−9^, 3.0×10^−11^, 2.5×10^−6^, 3.9×10^−10^, for places of articulation, manners of articulation, syllables and morpheme, respectively). **b**. Relationship between recording depth along the cortical column and the proportion of neurons that display selectivity to specific phonemes, syllables and morphemes. Neurons that displayed selectivity were broadly distributed along the cortical column (one-sided χ^2^ test, p > 0.05). Here, S indicates superficial, M middle and D deep.

Neurons that encoded information about the sublexical components of the upcoming words were broadly distributed across the cortex and cortical column depth. By tracking the location of each neuron in relation to the Neuropixels arrays, we find that there was a slightly higher preponderance of neurons that were tuned to phonemes (one-sided *χ*^2^ test (2) = 0.7 and 5.2, *P* > 0.05, for places and manners of articulation, respectively), syllables (one-sided *χ*^2^ test (2) = 3.6, *P* > 0.05) and morphemes (one-sided *χ*^2^ test (2) = 4.9, *P* > 0.05) at lower cortical depths, but that this difference was non-significant, suggesting a broad distribution (Extended Data Fig. 7). We also find, however, that the proportion of neurons that showed selectivity for phonemes increased as recordings were acquired more posteriorly along the rostral–caudal axis of the cortex (one-sided *χ*^2^ test (4) = 45.9 and 52.2, *P* < 0.01, for places and manners of articulation, respectively). Similar findings were also made for syllables and morphemes (one-sided *χ*^2^ test (4) = 31.4 and 49.8, *P* < 0.01, respectively; Extended Data Fig. 7); together suggesting a gradation of cellular representations, with caudal areas showing progressively higher proportions of selective neurons.

**Fig. 3:**
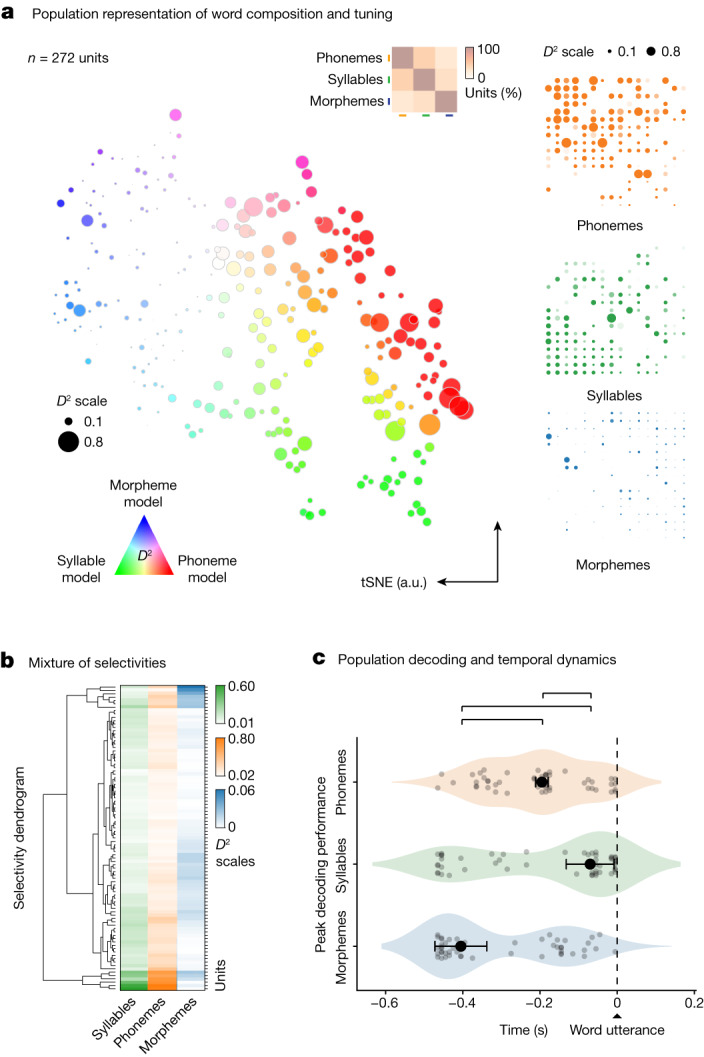
Temporal structure and organization of phonetic, syllabic and morphological representations. **a**, Left, response selectivity of neurons to specific word features (phonemes, syllables and morphemes) is visualized across the population using a tSNE procedure (that is, neurons with similar response characteristics were plotted in closer proximity). The hue of each point reflects the degree of selectivity to a particular sublexical feature whereas the size of each point reflects the degree to which those features explained neuronal response. Inset, the relative proportions of neurons showing selectivity and their overlap. Right, the *D*^2^ metric (the degree to which specific features explained neuronal response) for each cell shown individually per feature. **b**, The relative degree to which the activities of the neurons were explained by the phonetic, syllabic and morphological features of the words (*D*^2^ metric) and their hierarchical structure (agglomerative hierarchical clustering). **c**, Distribution of peak decoding performances for phonemes, syllables and morphemes aligned to word utterance onset. Significant differences in peak decoding timings across sample distribution are labelled in brackets above (*n* = 50 random test/train splits; *P* = 0.024, 0.002 and 0.002; pairwise, two-sided permutation tests of differences in medians for phonemes versus syllables, syllables versus morphemes and phonemes versus morphemes, respectively; Methods). Data are presented as median (dot) values ± bootstrapped standard error of the median. Source Data

Collectively, the activities of these cell ensembles provided richly detailed information about the phonetic, syllabic and morphological components of upcoming words. Of the neurons that showed selectivity to any sublexical feature, 51% (*n* = 46 of 90 units) were significantly informative of more than one feature. Moreover, the selectivity of these neurons lay along a continuum and were closely correlated (two-sided test of Pearson’s correlation in *D*^2^ across all sublexical feature comparisons, *r* = 0.80, 0.51 and 0.37 for phonemes versus syllables, phonemes versus morphemes and syllables versus morphemes, respectively, all *P* < 0.001; Fig. 2b), with most cells exhibiting a mixture of representations for specific phonetic, syllabic or morphological features (two-sided Wilcoxon signed-rank test, *P* < 0.0001). Figure 3a further illustrates this mixture of representations (Fig. 3a, left; *t*-distributed stochastic neighbour embedding (tSNE)) and their hierarchical structure (Fig. 3a, right; *D*^2^ distribution), together revealing a detailed characterization of the phonetic, syllabic and morphological components of upcoming words at the level of the cell population.

## Temporal organization of representations

**Extended Data Fig. 8:**
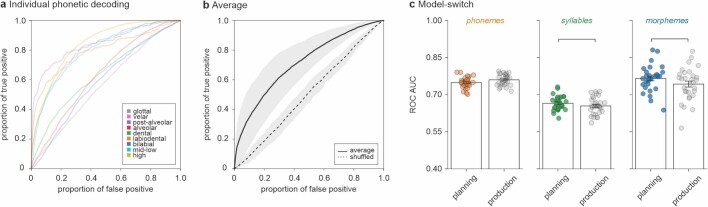
Receiver operating characteristic curves across planned phonetic representations and decoding model-switching performances for word planning and production. **a**. ROC-AUC curves for neurons across different phonemes, grouped by placed of articulation, during planning (there were insufficient palatal consonants to allow for classification and are therefore not displayed here). **b**. Average (solid line) and shuffled (dotted line) data across all phonemes. Data are presented as mean (line) values ± standard error of the mean (shade). **c**. Planning-production model-switch decoding performance sample distribution (n = 50 random test/train splits) for all selective neurons. Here, models were trained on neuronal data recorded during planning and then used to decode those same phoneme (*left*), syllable (*middle*), or morpheme (*right*) on neuronal data recorded during production. Slightly lower decoding performances were noted for syllables and morphemes when comparing word planning to production (p = 0.020 for syllable comparison and p = 0.032 for morpheme comparison, two-sided Mann–Whitney U-test). Data are presented as mean values ± standard deviation.

Given the above observations, we examined the temporal dynamic of neuronal activities during the production of speech. By tracking peak decoding in the period leading up to utterance onset (peak AUC; 50 model testing/training splits)^64^, we find these neural populations showed a consistent morphological–phonetic–syllabic dynamic in which decoding performance first peaked for morphemes. Peak decoding then followed for phonemes and syllables (Fig. 3b and Extended Data Fig. 8a,b; section on ‘Population modelling’). Overall, decoding performance peaked for the morphological properties of words at −405 ± 67 ms before utterance, followed by peak decoding for phonemes at −195 ± 16 ms and syllables at −70 ± 62 ms (s.e.m.; Fig. 3b). This temporal dynamic was highly unlikely to have been observed by chance (two-sided Kruskal–Wallis test, *H* = 13.28, *P* < 0.01) and was largely distinct from that observed during listening (two-sided Kruskal–Wallis test, *H* = 14.75, *P* < 0.001; Extended Data Fig. 6c). The activities of these neurons therefore seemed to follow a consistent, temporally ordered morphological–phonetic–syllabic dynamic before utterance.

**Fig. 4:**
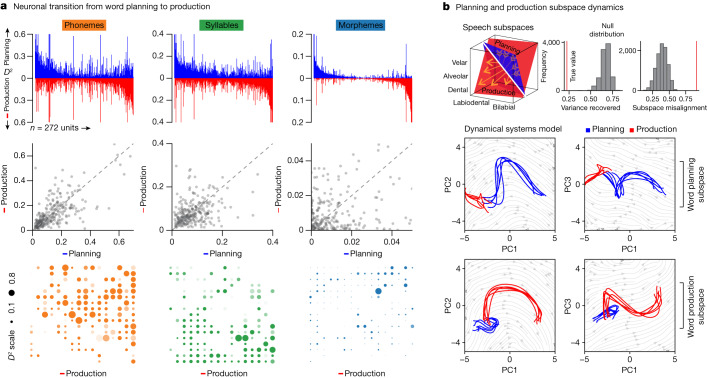
Neuronal population transition from articulation planning to production. **a**, Top, the *D*^2^ value of neuronal activity (the degree to which specific features explained neuronal response, *n* = 272 units) during word planning (green) and production (orange) sorted across all population neurons. Middle, relationship between explanatory power (*D*^2^) of neuronal activity (*n* = 272 units) for phonemes (Spearman’s *ρ* = 0.69), syllables (Spearman’s *ρ* = 0.40) and morphemes (Spearman’s *ρ* = 0.08) during planning and production (*P* = 1.3 × 10^−39^, *P* = 6.6 × 10^−12^, *P* = 0.18, respectively, two-sided test of Spearman rank-order correlation). Bottom, the *D*^2^ metric for each cell during production per feature (*n* = 272 units). **b**, Top left, schematic illustration of speech planning (blue plane) and production (red plane) subspaces as traversed by a neuron for different phonemes (yellow arrows; Extended Data Fig. 9). Top right, subspace misalignment quantified by an alignment index (red) or Grassmannian chordal distance (red) compared to that expected from chance (grey), demonstrating that the subspaces occupied by the neural population (*n* = 272 units) during planning and production were distinct. Bottom, projection of neural population activity (*n* = 272 units) during word planning (blue) and production (red) onto the first three PCs for the planning (upper row) and production (lower row) subspaces. Source Data

The activities of these neurons also followed a temporally structured transition from articulation planning to production. When comparing their activities before utterance onset (−500:0 ms) to those after (0:500 ms), we find that neurons which encoded information about the upcoming phonemes during planning encoded similar information during production (*P* < 0.001, Mann–Whitney *U*-test for phonemes and syllables; Fig. 4a). Moreover, when using models that were originally trained on words before utterance onset to decode the properties of the articulated words during production (model-switch approach), we find that decoding accuracy for the phonetic, syllabic and morphological properties of the words all remained significant (AUC = 0.76 ± 0.02 versus 0.48 ± 0.03 chance, 0.65 ± 0.03 versus 0.51 ± 0.04 chance, 0.74 ± 0.06 versus 0.44 ± 0.07 chance, for phonemes, syllables and morphemes, respectively; *P* < 0.001 for all, two-sided Mann–Whitney *U*-tests; Extended Data Fig. 8c). Information about the sublexical features of words was therefore reliably represented during articulation planning and execution by the neuronal population.

**Extended Data Fig. 9:**
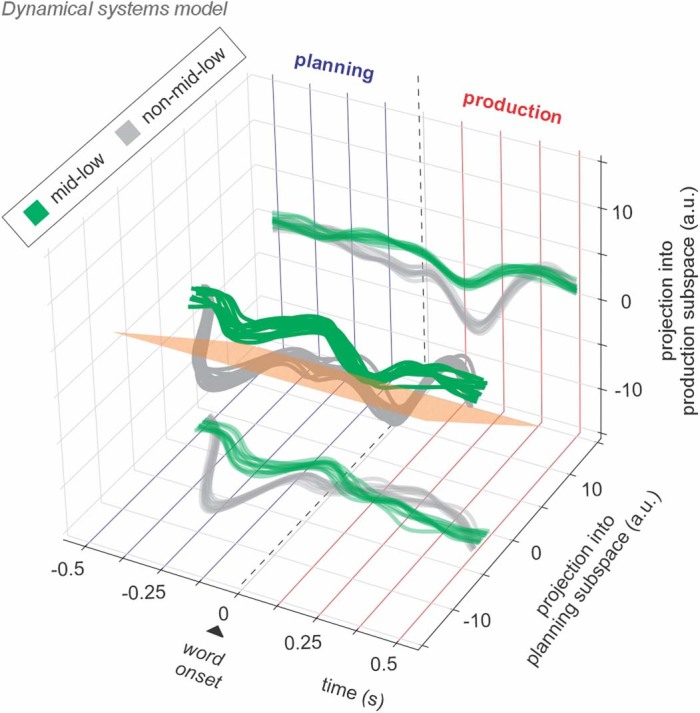
Example of phonetic representations in planning and production subspaces. Modelled depiction of the neuronal population trajectory (bootstrap resampled) across averaged trials with (green) and without (grey) mid-low phonemes, projected into a plane within the “planning” subspace (y-axis) and a plane within the “production” subspace (z-axis). Projection planes within planning and production subspaces were chosen to enable visualization of trajectory divergence. Zero indicates word onset on the x-axis. Separation between the population trajectory during trials with and without mid-low phonemes is apparent in the planning subspace (y-axis) independently of the projection subspace (z-axis) because these subspaces are orthogonal. The orange plane indicates a hypothetical decision boundary learned by a classifier to separate neuronal activities between mid-low and non-mid-low trials. Because the classifier decision boundary is not constrained to lie within a particular subspace, classifier performance may therefore generalize across planning and production epochs, despite the near-orthogonality of these respective subspaces.

Utilizing a dynamical systems approach to further allow for the unsupervised identification of functional subspaces (that is, wherein neural activity is embedded into a high-dimensional vector space; Fig. 4b, left; section on ‘Dynamical system and subspace analysis’)^31,34,65,66^, we find that the activities of the population were mostly low-dimensional, with more than 90% of the variance in neuronal activity being captured by its first four principal components (Fig. 4b, right). However, when tracking how the dimensions in which neural populations evolved over time, we also find that the subspaces which defined neural activity during articulation planning and production were largely distinct. In particular, whereas the first five subspaces captured 98.4% of variance in the trajectory of the population during planning, they captured only 11.9% of variance in the trajectory during articulation (two-sided permutation test, *P* < 0.0001; Fig. 4b, bottom and Extended Data Fig. 9). Together, these cell ensembles therefore seemed to occupy largely separate preparatory and motoric subspaces while also allowing for information about the phonetic, syllabic and morphological contents of the words to be stably represented during the production of speech.

## Discussion

Using Neuropixels probes to obtain acute, fine-scaled recordings from single neurons in the language-dominant prefrontal cortex^3–6^—in a region proposed to be involved in word planning^3–12^ and production^13–16^—we find a strikingly detailed organization of phonetic representations at a cellular level. In particular, we find that the activities of many of the neurons closely mirrored the way in which the word sounds were produced, meaning that they reflected how individual planned phonemes were generated through specific articulators^58,59^. Moreover, rather than simply representing phonemes independently of their order or structure, many of the neurons coded for their composition in the upcoming words. They also reliably predicted the arrangement and segmentation of phonemes into distinct syllables, together suggesting a process that could allow the structure and order of articulatory events to be encoded at a cellular level.

Collectively, this putative mechanism supports the existence of context-general representations of classes of speech sounds that speakers use to construct different word forms. In contrast, coding of sequences of phonemes as syllables may represent a context-specific representation of these speech sounds in a particular segmental context. This combination of context-general and context-specific representation of speech sound classes, in turn, is supportive of many speech production models which suggest that speakers hold abstract representations of discrete phonological units in a context-general way and that, as part of speech planning, these units are organized into prosodic structures that are context-specific^1,30^. Although the present study does not reveal whether these representations may be stored in and retrieved from a mental syllabary^1^ or are constructed from abstract phonology ad hoc, it lays a groundwork from which to begin exploring these possibilities at a cellular scale. It also expands on previous observations in animal models such as marmosets^67,68^, singing mice^69^ and canaries^70^ on the syllabic structure and sequence of vocalization processes, providing us with some of the earliest lines of evidence for the neuronal coding of vocal-motor plans.

Another interesting finding from these studies is the diversity of phonetic feature representations and their organization across cortical depth. Although our recordings sampled locally from relatively small columnar populations, most phonetic features could be reliably decoded from their collective activities. Such findings suggest that phonetic information necessary for constructing words may be potentially fully represented in certain regions along the cortical column^10,46–50^. They also place these populations at a putative intersection for the shared coding of places and manners of articulation and demonstrate how these representations may be locally distributed. Such redundancy and accessibility of information in local cortical populations is consistent with that observed from animal models^31–35^ and could serve to allow for the rapid orchestration of neuronal processes necessary for the real-time construction of words; especially during the production of natural speech. Our findings are also supportive of a putative ‘mirror’ system that could allow for the shared representation of phonetic features within the population when speaking and listening and for the real-time feedback of phonetic information by neurons during perception^23,71^.

A final notable observation from these studies is the temporal succession of neuronal encoding events. In particular, our findings are supportive of previous neurolinguistic theories suggesting closely coupled processes for coordination planned articulatory events that ultimately produces words. These models, for example, suggest that the morphology of a word is probably retrieved before its phonologic code, as the exact phonology depends on the morphemes in the word form^1^. They also suggest the later syllabification of planned phonemes which would enable them to be sequentially arranged in specific order (although different temporal orders have been suggested as well)^72^. Here, our findings provide tentative support for a structured sublexical coding succession that could allow for the discretization of such information during articulation. Our findings also suggest (through dynamical systems modelling) a mechanism that, consistent with previous observations on motor planning and execution^31,34,65,66^, could enable information to occupy distinct functional subspaces^34,73^ and therefore allow for the rapid separation of neural processes necessary for the construction and articulation of words.

Taken together, these findings reveal a set of processes and framework in the language-dominant prefrontal cortex by which to begin understanding how words may be constructed during natural speech at a single-neuronal level through which to start defining their fine-scale spatial and temporal dynamics. Given their robust decoding performances (especially in the absence of natural language processing-based predictions), it is interesting to speculate whether such prefrontal recordings could also be used for synthetic speech prostheses or for the augmentation of other emerging approaches^21,22,74^ used in brain–machine interfaces. It is important to note, however, that the production of words also involves more complex processes, including semantic retrieval, the arrangement of words in sentences, and prosody, which were not tested here. Moreover, future experiments will be required to investigate eloquent areas such as ventral premotor and superior posterior temporal areas not accessible with our present techniques. Here, this study provides a prospective platform by which to begin addressing these questions using a combination of ultrahigh-density microelectrode recordings, naturalistic speech tracking and acute real-time intraoperative neurophysiology to study human language at cellular scale.

## Methods

### Study participants

All aspects of the study were carried out in strict accordance with and were approved by the Massachusetts General Brigham Institutional Review Board. Right-handed native English speakers undergoing awake microelectrode recording-guided deep brain stimulator implantation were screened for enrolment. Clinical consideration for surgery was made by a multidisciplinary team of neurosurgeons, neurologists and neuropsychologists. Operative planning was made independently by the surgical team and without consideration of study participation. Participants were only enroled if: (1) the surgical plan was for awake microelectrode recording-guided placement, (2) the patient was at least 18 years of age, (3) they had intact language function with English fluency and (4) were able to provide informed consent for study participation. Participation in the study was voluntary and all participants were informed that they were free to withdraw from the study at any time.

### Acute intraoperative single-neuronal recordings

#### Single-neuronal prefrontal recordings using Neuropixels probes

As part of deep brain stimulator implantation at our institution, participants are often awake and microelectrode recordings are used to optimize anatomical targeting of the deep brain structures^46^. During these cases, the electrodes often traverse part of the posterior language-dominant prefrontal cortex^3–6^ in an area previously shown be involved in word planning^3–12^ and sentence construction^13–16^ and which broadly connects with premotor areas involved in their articulation^51–53^ and lexical processing^17–19^ by imaging studies (Extended Data Fig. 1a,b). All microelectrode entry points and placements were based purely on planned clinical targeting and were made independently of any study consideration.

Sterile Neuropixels probes (v.1.0-S, IMEC, ethylene oxide sterilized by BioSeal^54^) together with a 3B2 IMEC headstage were attached to cannula and a manipulator connected to a ROSA ONE Brain (Zimmer Biomet) robotic arm. Here, the probes were inserted into the cortical ribbon under direct robot navigational guidance through the implanted burr hole (Fig. 1a). The probes (width 70 µm; length 10 mm; thickness 100 µm) consisted of a total of 960 contact sites (384 preselected recording channels) laid out in a chequerboard pattern with approximately 25 µm centre-to-centre nearest-neighbour site spacing. The IMEC headstage was connected through a multiplexed cable to a PXIe acquisition module card (IMEC), installed into a PXIe Chassis (PXIe-1071 chassis, National Instruments). Neuropixels recordings were performed using SpikeGLX (v.20201103 and v.20221012-phase30; http://billkarsh.github.io/SpikeGLX/) or OpenEphys (v.0.5.3.1 and v.0.6.0; https://open-ephys.org/) on a computer connected to the PXIe acquisition module recording the action potential band (AP, band-pass filtered from 0.3 to 10 kHz) sampled at 30 kHz and a local-field potential band (LFP, band-pass filtered from 0.5 to 500 Hz), sampled at 2,500 Hz. Once putative units were identified, the Neuropixels probe was briefly held in position to confirm signal stability (we did not screen putative neurons for speech responsiveness). Further description of this recording approach can be found in refs. ^54,55^. After single-neural recordings from the cortex were completed, the Neuropixels probe was removed and subcortical neuronal recordings and deep brain stimulator placement proceeded as planned.

#### Single-unit isolation

Single-neuronal recordings were performed in two main steps. First, to track the activities of putative neurons at high spatiotemporal resolution and to account for intraoperative cortical motion, we use a Decentralized Registration of Electrophysiology Data software (DREDge; https://github.com/evarol/DREDge) and interpolation approach (https://github.com/williamunoz/InterpolationAfterDREDge). Briefly, and as previously described^54–56^, an automated protocol was used to track LFP voltages using a decentralized correlation technique that re-aligned the recording channels in relation to brain movements (Fig. 1a, right). Following this step, we then interpolated the AP band continuous voltage data using the DREDge motion estimate to allow the activities of the putative neurons to be stably tracked over time. Next, single units were isolated from the motion-corrected interpolated signal using Kilosort (v.1.0; https://github.com/cortex-lab/KiloSort) followed by Phy for cluster curation (v.2.0a1; https://github.com/cortex-lab/phy; Extended Data Fig. 1c,d). Here, units were selected on the basis of their waveform morphologies and separability in principal component space, their interspike interval profiles and similarity of waveforms across contacts. Only well-isolated single units with mean firing rates ≥0.1 Hz were included. The range of units obtained from these recordings was 16–115 units per participant.

### Audio recordings and task synchronization

For task synchronization, we used the TTL output and audio output to send the synchronization trigger through the SMA input to the IMEC PXIe acquisition module card. To allow for added synchronizing, triggers were also recorded on an extra breakout analogue and digital input/output board (BNC2110, National Instruments) connected through a PXIe board (PXIe-6341 module, National Instruments).

Audio recordings were obtained at 44 kHz sampling frequency (TASCAM DR-40×4-Channel/ 4-Track Portable Audio Recorder and USB Interface with Adjustable Microphone) which had an audio input. These recordings were then sent to a NIDAQ board analogue input in the same PXIe acquisition module containing the IMEC PXIe board for high-fidelity temporal alignment with neuronal data. Synchronization of neuronal activity with behavioural events was performed through TTL triggers through a parallel port sent to both the IMEC PXIe board (the sync channel) and the analogue NIDAQ input as well as the parallel audio input into the analogue input channels on the NIDAQ board.

Audio recordings were annotated in semi-automated fashion (Audacity; v.2.3). Recorded audio for each word and sentence by the participants was analysed in Praat^75^ and Audacity (v.2.3). Exact word and phoneme onsets and offsets were identified using the Montreal Forced Aligner (v.2.2; https://github.com/MontrealCorpusTools/Montreal-Forced-Aligner)^76^ and confirmed with manual review of all annotated recordings. Together, these measures allowed for the millisecond-level alignment of neuronal activity with each produced word and phoneme.

### Anatomical localization of recordings

Pre-operative high-resolution magnetic resonance imaging and postoperative head computerized tomography scans were coregistered by combination of ROSA software (Zimmer Biomet; v.3.1.6.276), Mango (v.4.1; https://mangoviewer.com/download.html) and FreeSurfer (v.7.4.1; https://surfer.nmr.mgh.harvard.edu/fswiki/DownloadAndInstall) to reconstruct the cortical surface and identify the cortical location from which Neuropixels recordings were obtained^77–81^. This registration allowed localization of the surgical areas that underlaid the cortical sites of recording (Fig. 1a and Extended Data Fig. 1a)^54–56^. The MNI transformation of these coordinates was then carried out to register the locations in MNI space with Fieldtrip toolbox (v.20230602; https://www.fieldtriptoolbox.org/; Extended Data Fig. 1b)^82^.

For depth calculation, we estimated the pial boundary of recordings according to the observed sharp signal change in signal from channels that were implanted in the brain parenchyma versus those outside the brain. We then referenced our single-unit recording depth (based on their maximum waveform amplitude channel) in relation to this estimated pial boundary. Here, all units were assessed on the basis of their relative depths in relation to the pial boundary as superficial, middle and deep (Extended Data Fig. 7).

### Speech production task

The participants performed a priming-based naturalistic speech production task^57^ in which they were given a scene on a screen that consisted of a scenario that had to be described in specific order and format. Thus, for example, the participant may be given a scene of a boy and a girl playing with a balloon or they may be given a scene of a dog chasing a cat. These scenes, together, required the participants to produce words that varied in phonetic, syllabic and morphosyntactic content. They were also highlighted in a way that required them to produce the words in a structured format. Thus, for example, a scene may be highlighted in a way that required the participants to produce the sentence “The mouse was being chased by the cat” or in a way that required them to produce the sentence “The cat was chasing the mouse” (Extended Data Fig. 2a). Because the sentences had to be constructed de novo, it also required the participants to produce the words without providing explicit phonetic cues (for example, from hearing and then repeating the word ‘cat’). Taken together, this task therefore allowed neuronal activity to be examined whereby words (for example, ‘cat’), rather than independent phonetic sounds (for example, /k/), were articulated and in which the words were produced during natural speech (for example, constructing the sentence “the dog chased the cat”) rather than simply repeated (for example, hearing and then repeating the word ‘cat’).

Finally, to account for the potential contribution of sensory–perceptual responses, three of the participants also performed a ‘perception’ control in which they listened to words spoken to them. One of these participants further performed an auditory ‘playback’ control in which they listened to their own recorded voice. For this control, all words spoken by the participant were recorded using a high-fidelity microphone (Zoom ZUM-2 USM microphone) and then played back to them on a word-by-word level in randomized separate blocks.

### Constructing a word feature space

#### Phonemes

To allow for single-neuronal analysis and to provide a compositional representation for each word, we grouped the constituent phonemes on the basis of the relative positions of articulatory organs associated with their production^60^. Here, for our primary analyses, we selected the places of articulation for consonants (for example, bilabial consonants) on the basis of established IPA categories defining the primary articulators involved in speech production. For consonants, phonemes were grouped on the basis of their places of articulation into glottal, velar, palatal, postalveolar, alveolar, dental, labiodental and bilabial. For vowels, we grouped phonemes on the basis of the relative height of the tongue with high vowels being produced with the tongue in a relatively high position and mid-low (that is, mid+low) vowels being produced with it in a lower position. Here, this grouping of phonemes is broadly referred to as ‘places of articulation’ together reflecting the main positions of articulatory organs and their combinations used to produce the words^58,59^. Finally, to allow for comparison and to test their generalizability, we examined the manners of articulation stop, fricative, affricate, nasal, liquid and glide for consonants which describe the nature of airflow restriction by various parts of the mouth and tongue. For vowels, we also evaluated the primary cardinal vowels i, e, ɛ, a, α, ɔ, o and u which are described, in combination, by the position of the tongue relative to the roof of the mouth, how far forward or back it lies and the relative positions of the lips^83,84^. A detailed summary of these phonetic groupings can be found in Extended Data Table 1.

#### Phoneme feature space

To further evaluate the relationship between neuronal activity and the presence of specific constituent phonemes per word, the phonemes in each word were parsed according to their precise pronunciation provided by the English Lexicon Project (or the Longman Pronunciation Dictionary for American English where necessary) as described previously^85^. Thus, for example, the word ‘like’ (l-aɪ-k) would be parsed into a sequence of alveolar-mid-low-velar phonemes, whereas the word ‘bike’ (b-aɪ-k) would be parsed into a sequence of bilabial-mid-low-velar phonemes.

These constituent phonemes were then used to represent each word as a ten-dimensional vector in which the value in each position reflected the presence of each type of phoneme (Fig. 1c). For example, the word ‘like’, containing a sequence of alveolar-mid-low-velar phonemes, was represented by the vector [0 0 0 1 0 0 1 0 0 1], with each entry representing the number of the respective type of phoneme in the word. Together, such vectors representing all words defined a phonetic ‘vector space’. Further analyses to evaluate the precise arrangement of phonemes per word are described further below. Goodness-of-fit and selectivity metrics used to evaluate single-neuronal responses to these phonemes and their specific combination in words are described further below.

#### Syllabic feature space

Next, to evaluate the relationship between neuronal activity and the specific arrangement of phonemes in syllables, we parsed the constituent syllables for each word using American pronunciations provided in ref. ^85^. Thus, for example, ‘back’ would be defined as a labial-low-velar sequence. Here, to allow for neuronal analysis and to limit the combination of all possible syllables, we selected the ten most common syllable types. High and mid-low vowels were considered as syllables here only if they reflected syllables in themselves and were unbound from a consonant (for example, /ih/ in ‘hesitate’ or /ah-/ in ‘adore’). Similar to the phoneme space, the syllables were then transformed into an *n*-dimensional binary vector in which the value in each dimension reflected the presence of specific syllables (similar to construction of the phoneme space). Thus, for the *n*-dimensional representation of each word in this syllabic feature space, the value in each dimension could be also interpreted in relation to neuronal activity.

#### Morphemes

To account for the functional distinction between phonemes and morphemes^62,63^, we also parsed words into those that contained bound morphemes which were either prefixed (for example, ‘re–’) or suffixed (for example, ‘–ed’). Unlike phonemes, morphemes such as ‘–ed’ in ‘directed’ or ‘re–’ in ‘retry’ are the smallest linguistic units capable of carrying meaning and, therefore, accounting for their presence allowed their effect on neuronal responses to be further examined. To allow for neuronal analysis and to control for potential differences in neuronal activity due to word lengths, models also took into account the total number of phonemes per word.

#### Spectral features

To evaluate the time-varying spectral features of the articulated phonemes on a phoneme-by-phoneme basis, we identified the occurrence of each phoneme using a Montreal Forced Aligner (v.2.2; https://github.com/MontrealCorpusTools/Montreal-Forced-Aligner). For pitch, we calculated the spectral power in ten log-spaced frequency bins from 200 to 5,000 Hz for each phoneme per word. For amplitude, we took the root-mean-square of the recorded waveform of each phoneme.

### Single-neuronal analysis

#### Evaluating the selectivity of single-neuronal responses

To investigate the relationship between single-neuronal activity and specific word features, we used a regression analysis to determine the degree to which variation in neural activity could be explained by phonetic, syllabic or morphologic properties of spoken words^86–89^. For all analyses, neuronal activity was considered in relation to word utterance onset (*t* = 0) and taken as the mean spike count in the analysis window of interest (that is, −500 to 0 ms from word onset for word planning and 0 to +500 ms for word production). To limit the potential effects of preceding words on neuronal activity, words with planning periods that overlapped temporally were excluded from regression and selectivity analyses. For each neuron, we constructed a GLM that modelled the spike count rate as the realization of a Poisson process whose rate varied as a function of the linguistic (for example, phonetic, syllabic and morphologic) or acoustic features (for example, spectral power and root-mean-square amplitude) of the planned words.

Models were fit using the Python (v.3.9.17) library statsmodels (v.0.13.5) by iterative least-squares minimization of the Poisson negative log-likelihood function^86^. To assess the goodness-of-fit of the models, we used both the Akaike information criterion (${\rm{AIC}}=2k-2{\rm{ln}}(L)$ where *k* is the number of estimated parameters and *L* is the maximized value of the likelihood function) and a generalization of the *R*^2^ score for the exponential family of regression models that we refer to as *D*^2^ whereby^87^:

$${D}^{2}=1-\frac{K({\bf{y}},{{\boldsymbol{\mu }}}_{{\rm{full}}})}{K({\bf{y}},{{\boldsymbol{\mu }}}_{{\rm{restricted}}})}$$

**y** is a vector of realized outcomes, **μ** is a vector of estimated means from a full (including all regressors) or restricted (without regressors of interest) model and ${K}({\bf{y}}\,,{\boldsymbol{\mu }})=2\bullet {\rm{llf}}({\bf{y}}\,;{\bf{y}})-2\bullet {\rm{llf}}({\boldsymbol{\mu }}\,;{\bf{y}})$ where ${\rm{llf}}({\boldsymbol{\mu }}\,;{\bf{y}})$ is the log-likelihood of the model and ${\rm{llf}}({\bf{y}}\,;{\bf{y}})$ is the log-likelihood of the saturated model. The *D*^2^ value represents the proportion of reduction in uncertainty (measured by the Kullback–Leibler divergence) due to the inclusion of regressors. The statistical significance of model fit was evaluated using the likelihood ratio test compared with a model with all covariates except the regressors of interest (the task variables).

We characterized a neuron as selectively ‘tuned’ to a given word feature if the GLM of neuronal firing rates as a function of task variables for that feature exhibited a statistically significant model fit (likelihood ratio test with *α* set at 0.01). For neurons meeting this criterion, we also examined the point estimates and confidence intervals for each coefficient in the model. A vector of these coefficients (or, in our feature space, a vector of the sign of these coefficients) indicates a word with the combination of constituent elements expected to produce a maximal neuronal response. The multidimensional feature spaces also allowed us to define metrics that quantified the phonemic, syllabic or morphologic similarity between words. Here, we calculated the Hamming distance between the vector describing each word **u** and the vector of the sign of regression coefficients that defines each neuron’s maximal predicted response **v**, which is equal to the number of positions at which the corresponding values are different:

$${\rm{Hamming}}\,{\rm{distance}}={\rm{count}}\left\{i:{{\bf{u}}}_{i}\ne {{\bf{v}}}_{i},i=1\ldots n\right\}$$

For each ‘tuned’ neuron, we compared the *Z*-scored firing rate elicited by each word as a function of the Hamming distance between the word and the ‘preferred word’ of the neuron to examine the ‘tuning’ characteristics of these neurons (Figs. 1f and 2c). A Hamming distance of zero would therefore indicate that the words have phonetically identical compositions. Finally, to examine the relationship between neuronal activity and spectral features of each phoneme, we extracted the acoustic waveform for each phoneme and calculated the power in ten log-spaced spectral bands. We then constructed a ‘spectral vector’ representation for each word based on these ten values and fit a Poisson GLM of neuronal firing rates against these values. For amplitude analysis, we regressed neuronal firing rates against the root-mean-square amplitude of the waveform for each word.

#### Controlling for interdependency between phonetic and syllabic features

Three more word variations were used to examine the interdependency between phonetic and syllabic features. First, we compared firing rates for words containing specific syllables with words containing individual phonemes in that syllable but not the syllable itself (for example, simply /d/ in ‘god’ or ‘dog’). Second, we examined words containing syllables with the same constituent phonemes but in a different order (for example, /g-ah-d/ for ‘god’ versus /d-ah-g/ for ‘dog’). Thus, if neurons responded preferentially to specific syllables, then they should continue to respond to them preferentially even when comparing words that had the same arrangements of phonemes but in different or reverse order. Third, we examined words containing the same sequence of syllables but spanning a syllable boundary such that the cluster of phonemes did not constitute a syllable (that is, in the same syllable versus spanning across syllable boundaries).

#### Visualization of neuronal responses within the population

To allow for visualization of groupings of neurons with shared representational characteristics, we calculated the AIC and *D*^2^ for phoneme, syllable and morpheme models for each neuron and conducted tSNE procedure which transformed these data into two dimensions such that neurons with similar feature representations are spatially closer together than those with dissimilar representations^90^. We used the tSNE implantation in the scikit-learn Python module (v.1.3.0). In Fig. 3a left, a tSNE was fit on the AIC values for phoneme, syllable and morpheme models for each neuron during the planning period with the following parameters: perplexity = 35, early exaggeration = 2 and using Euclidean distance as the metric. In Fig. 3a right and Fig. 4a bottom, a different tSNE was fit on the *D*^2^ values for all planning and production models using the following parameters: perplexity = 10, early exaggeration = 10 and using a cosine distance metric. The resulting embeddings were mapped onto a grid of points according to a linear sum assignment algorithm between embeddings and grid points.

### Population modelling

#### Modelling population activity

To quantify the degree to which the neural population coded information about the planned phonemes, syllables and morphemes, we modelled the activity of the entire pseudopopulation of recorded neurons. To match trials across the different participants, we first labelled each word according to whether it contained the feature of interest and then matched words across subjects based on the features that were shared. Using this procedure, no trials or neural data were duplicated or upsampled, ensuring strict separation between training and testing sets during classifier training and subsequent evaluation.

For decoding, words were randomly split into training (75%) and testing (25%) trials across 50 iterations. A support vector machine (SVM) as implemented in the scikit-learn Python package (v.1.3.0)^91^ was used to construct a hyperplane in *n*-dimensional space that optimally separates samples of different word features by solving the following minimization problem:

$$\min \left(\frac{1}{2}{w}^{T}w+C\mathop{\sum }\limits_{i=1}^{n}{\zeta }_{i}\right)$$

subject to ${y}_{i}({w}^{T}\phi ({x}_{i})+b)\ge 1-{\zeta }_{i}$ and ${\zeta }_{i}\ge 0$ for all $i\in \left\{1,\ldots ,n\right\}$, where *w* is the margin in feature space, *C* is the regularization strength, *ζ*_*i*_ is the distance of each point from the margin, *y*_*i*_ is the predicted class for each sample and *ϕ*(*x*_*i*_) is the image of each datapoint in transformed feature space. A radial basis function kernel with coefficient *γ* = 1/272 was applied. The penalty term *C* was optimized for each classifier using a cross-validation procedure nested in the training set.

A separate classifier was trained for each dimension in a task space (for example, separate classifiers for bilabial, dental and alveolar consonants) and scores for each of these classifiers were averaged to calculate an overall decoding score for that feature type. Each decoder was trained to predict whether the upcoming word contained an instance of a specific phoneme, syllable or morpheme arrangement. For phonemes, we used nine of the ten phoneme groups (there were insufficient instances of palatal consonants to train a classifier; Extended Data Table 1). For syllables, we used ten syllables taken from the most common syllables across the study vocabulary (Extended Data Table 1). For morpheme analysis, a single classifier was trained to predict the presence or absence of any bound morpheme in the upcoming word.

Finally, to assess performance, we scored classifiers using the area under the curve of the receiver operating characteristic (AUC-ROC) model. With this scoring metric, a classifier that always guesses the most common class (that is, an uninformative classifier) results in a score of 0.5 whereas a perfect classification results in a score of 1. The overall decoding score for a particular feature space was the mean score of the classifier for each dimension in the space. The entire procedure was repeated 50 times with random train/test splits. Summary statistics for these 50 iterations are presented in the main text.

#### Model switching

Assessing decoder generalization across different experimental conditions provides a powerful method to evaluate the similarity of neuronal representations of information in different contexts^64^. To determine how neurons encoded the same word features but under different conditions, we trained SVM decoders using neuronal data during one condition (for example, word production) but tested the decoder using data from another (for example, no word production). Before decoder training or testing, trials were split into disjoint training and testing sets, from which the neuronal data were extracted in the epoch of interest. Thus, trials used to train the model were never used to test the model while testing either native decoder performance or decoder generalizability.

#### Modelling temporal dynamic

To further study the temporal dynamic of neuronal response, we trained decoders to predict the phonemes, syllables and morpheme arrangement for each word across successive time points before utterance^64^. For each neuron, we aligned all spikes to utterance onset, binned spikes into 5 ms windows and convolved with a Gaussian kernel with standard deviation of 25 ms to generate an estimated instantaneous firing rate at each point in time during word planning. For each time point, we evaluated the performance of decoders of phonemes, syllables and morphemes trained on these data over 50 random splits of training and testing trials. The distribution of times of peak decoding performance across the planning or perception period revealed the dynamic of information encoding by these neurons during word planning or perception and we then calculated the median peak decoding times for phonemes, syllables or morphemes.

### Dynamical system and subspace analysis

To study the dimensionality of neuronal activity and to evaluate the functional subspaces occupied by the neuronal population, we used dynamical systems approach that quantified the time-dependent changes in neural activity patterns^31^. For the dynamical system analysis, activity for all words were averaged for each neuron to come up with a single peri-event time projection (aligned to word onset) which allowed all neurons to be analysed together as a pseudopopulation. First, we calculated the instantaneous firing rates of the neuron which showed selectivity to any word feature (phonemes, syllables or morpheme arrangement) into 5 ms bins convolved with a Gaussian filter with standard deviation of 50 ms. We used equal 500 ms windows set at −500 to 0 ms before utterance onset for the planning phase and 0 to 500 ms following utterance onset for the production phase to allow for comparison. These data were then standardized to zero mean and unit variance. Finally, the neural data were concatenated into a *T* *×* *N* matrix of sampled instantaneous firing rates for each of the *N* neurons at every time *T*.

Together, these matrices represented the evolution of the system in *N*-dimensional space over time. A principal component analysis revealed a small set of five principal components (PC) embedded in the full *N*-dimensional space that captured most of the variance in the data for each epoch (Fig. 4b). Projection of the data into this space yields a *T* × 5 matrix representing the evolution of the system in five-dimensional space over time. The columns of the *N* × 5 principal components form an orthonormal basis for the five-dimensional subspace occupied by the system during each epoch.

Next, to quantify the relationship between these subspaces during planning and production, we took two approaches. First, we calculated the alignment index from ref. ^66^:

$$A=\frac{{\rm{Tr}}({D}_{{\rm{A}}}^{\intercal}{C}_{{\rm{B}}}{D}_{{\rm{A}}})}{{\sum }_{i}{\sigma }_{{\rm{B}}}(i)}$$

where *D*_A_ is the matrix defined by the orthonormal basis of subspace A, *C*_B_ is the covariance of the neuronal data as it evolves in space B, ${\sigma }_{{\rm{B}}}(i)$ is the *i*th singular value of the covariance matrix *C*_B_ and Tr(∙) is the matrix trace. The alignment index *A* ranges from 0 to 1 and quantifies the fraction of variance in space B recovered when the data are projected into space A. Higher values indicate that variance in the data is adequately captured by either subspace.

As discussed in ref. ^66^, subspace misalignment in the form of low alignment index A can arise by chance when considering high-dimensional neuronal data because of the probability that two randomly selected sets of dimensions in high-dimensional space may not align well. Therefore, to further explore the degree to which our subspace misalignment was attributable to chance, we used the Monte Carlo analysis to generate random subspaces from data with the same covariance structure as the true (observed) data:

$${\bf{V}}={\rm{orth}}\left(\frac{U\sqrt{S}\,v}{{\parallel U\sqrt{S}v\parallel }_{2}}\right)$$

where **V** is a random subspace, *U* and *S* are the eigenvectors and eigenvalues of the covariance matrix of the observed data across all epochs being compared, *v* is a matrix of white noise and orth(∙) orthogonalizes the matrix. The alignment index *A* of the subspaces defined by the resulting basis vectors **V** was recalculated 1,000 times to generate a distribution of alignment index values *A* attributable to chance alone (compare Fig. 4b).

Finally, we calculated the projection error between each pair of subspaces on the basis of relationships between the three orthonormal bases (rather than a projection of the data into each of these subspaces). The set of all (linear) subspaces of dimension *k* *<* *n* embedded in an *n*-dimensional vector space **V** forms a manifold known as the Grassmannian, endowed with several metrics which can be used to quantify distances between two subspaces on the manifold. Thus, the subspaces (defined by the columns of a *T* *×* *N*′ matrix, where *N*′ is the number of selected principal components; five in our case) explored by the system during planning and production are points on the Grassmannian manifold of the full *N*-neuron dimensional vector space. Here, we used the Grassmannian chordal distance^92^:

$${\rm{d}}(A,B)=\,\frac{1}{\sqrt{2}}{\parallel A{A}^{\intercal}-B{B}^{\intercal}\parallel }_{F}$$

where *A* and *B* are matrices whose columns are the orthonormal basis for their respective subspaces and ${\parallel \cdot \parallel }_{F}$ is the Frobenius norm. By normalizing this distance by the Frobenius norm of subspace *A*, we scale the distance metric from 0 to 1, where 0 indicates a subspace identical to *A* (that is, completely overlapping) and increasing values indicate greater misalignment from *A*. Random sampling of subspaces under the null hypothesis was repeated using the same procedure outlined above.

### Participant demographics

Across the participants, there was no statistically significant difference in word length based on sex (three-way analysis of variance, *F*(1,4257) = 1.78, *P* = 0.18) or underlying diagnosis (essential tremor versus Parkinson’s disease; *F*(1,4257) = 0.45, *P* = 0.50). Among subjects with Parkinson’s disease, there was a significant difference based on disease severity (both ON score and OFF score) with more advanced disease (higher scores) correlating with longer word lengths (*F*(1,3295) = 145.8, *P* = 7.1 × 10^−33^ for ON score and *F*(1,3295) = 1,006.0, *P* = 6.7 × 10^−193^ for OFF score, *P* < 0.001) and interword intervals (*F*(1,3291) = 14.9, *P* = 1.1 × 10^−4^ for ON score and *F*(1,3291) = 31.8, *P* = 1.9 × 10^−8^ for OFF score). Modelling neuronal activities in relation to these interword intervals (bottom versus top quartile), decoding performances were slightly higher for longer compared to shorter delays (0.76 ± 0.01 versus 0.68 ± 0.01, *P* < 0.001, two-sided Mann–Whitney *U*-test).

### Reporting summary

Further information on research design is available in the Nature Portfolio Reporting Summary linked to this article.

## Online content

Any methods, additional references, Nature Portfolio reporting summaries, source data, extended data, supplementary information, acknowledgements, peer review information; details of author contributions and competing interests; and statements of data and code availability are available at 10.1038/s41586-023-06982-w.

### Supplementary information

Reporting Summary

### Source data

Source Data Fig. 1

Source Data Fig. 2

Source Data Fig. 3

Source Data Fig. 4

## Figures and Tables

**Extended Data Table 1:**
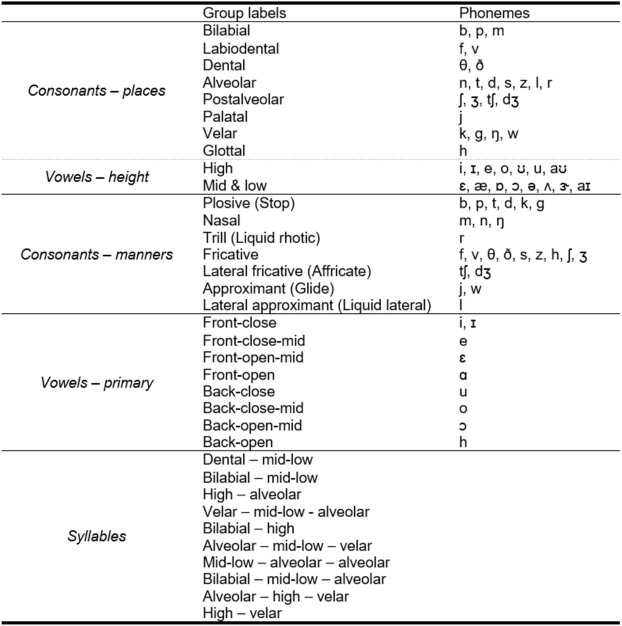
Phonetic groupings

To provide a compositional representation of each word for the main analyses, phonemes were grouped based on their places of articulation for consonants (for example, velar and bilabial) and relative height of the tongue for vowels (for example, high and mid-low). For comparison, phonemes were also grouped based on the manners of articulation (for example, plosive and fricative) as well as the combined position of the tongue and lips for vowels (for example, front-close or back-open). Finally, we used the ten most common syllables in the study’s vocabulary to study the words’ syllabic structures.

## Data Availability

All the primary data supporting the main findings of this study are available online at 10.6084/m9.figshare.24720501. Source data are provided with this paper.

## Code availability

All codes necessary for reproducing the main findings of this study are available online at 10.6084/m9.figshare.24720501.
