# Supplementary material for: Single-neuronal elements of speech production in humans
Source: Nature. 2024 Jan 31;626(7999):603–10. doi: 10.1038/s41586-023-06982-w (PMC10866697; doi:10.1038/s41586-023-06982-w)
Supplement: Supplementary file 1 — Reporting Summary [file 41586_2023_6982_MOESM1_ESM.pdf]

nature research

Corresponding author(s): Ziv Williams

Last updated by author(s): Dec 3, 2023

# Reporting Summary

Nature Research wishes to improve the reproducibility of the work that we publish. This form provides structure for consistency and transparency in reporting. For further information on Nature Research policies, see our Editorial Policies and the Editorial Policy Checklist.

## Statistics

For all statistical analyses, confirm that the following items are present in the figure legend, table legend, main text, or Methods section.

| n/a | Confirmed | |
|---|---|---|
| ☐ | ☒ | The exact sample size (*n*) for each experimental group/condition, given as a discrete number and unit of measurement |
| ☐ | ☒ | A statement on whether measurements were taken from distinct samples or whether the same sample was measured repeatedly |
| ☐ | ☒ | The statistical test(s) used AND whether they are one- or two-sided<br>*Only common tests should be described solely by name; describe more complex techniques in the Methods section.* |
| ☐ | ☒ | A description of all covariates tested |
| ☐ | ☒ | A description of any assumptions or corrections, such as tests of normality and adjustment for multiple comparisons |
| ☐ | ☒ | A full description of the statistical parameters including central tendency (e.g. means) or other basic estimates (e.g. regression coefficient) AND variation (e.g. standard deviation) or associated estimates of uncertainty (e.g. confidence intervals) |
| ☐ | ☒ | For null hypothesis testing, the test statistic (e.g. *F*, *t*, *r*) with confidence intervals, effect sizes, degrees of freedom and *P* value noted<br>*Give P values as exact values whenever suitable.* |
| ☒ | ☐ | For Bayesian analysis, information on the choice of priors and Markov chain Monte Carlo settings |
| ☐ | ☒ | For hierarchical and complex designs, identification of the appropriate level for tests and full reporting of outcomes |
| ☐ | ☒ | Estimates of effect sizes (e.g. Cohen's *d*, Pearson's *r*), indicating how they were calculated |

*Our web collection on statistics for biologists contains articles on many of the points above.*

## Software and code

Policy information about availability of computer code

| | |
|---|---|
| Data collection | SpikeGLX (release v20201103-phase30 and release_v20221012-phase30; http://billkarsh.github.io/SpikeGLX/) and OpenEphys (versions 0.5.3.1 and 0.6.0; https://open-ephys.org/gui) were employed. |
| Data analysis | Recording motion estimation was carried out with DREDge (https://github.com/evarol/dredge), motion correction with (https://github.com/williamunoz/InterpolationAfterDREDge), single-unit activity isolation with Kilosort (version 1.0; https://github.com/cortex-lab/KiloSort) and curation with Phy (version 2.0a1; https://github.com/cortex-lab/phy). Speech analysis was carried out with Audacity (version 2.3.0), and exact word and phoneme onsets and offsets were identified using the Montreal Forced Aligner (version 2.2; https://github.com/MontrealCorpusTools/Montreal-Forced-Aligner). Anatomical analysis was carried out with ROSA (Zimmer Biomet, version 3.1.6.276), Mango (version 4.1; https://mangoviewer.com/download.html), FreeSurfer (version 7.4.1; https://surfer.nmr.mgh.harvard.edu/fswiki/DownloadAndInstall) and Fieldtrip (version 20230602; https://www.fieldtriptoolbox.org/). Python (version 3.9.17; statsmodels version 0.13.5, scikit-learn module version 1.3.0) and Matlab (version R2023a) custom routines were employed for neuronal activity analysis and visualization, as well as statistical analysis. |

For manuscripts utilizing custom algorithms or software that are central to the research but not yet described in published literature, software must be made available to editors and reviewers. We strongly encourage code deposition in a community repository (e.g. GitHub). See the Nature Research guidelines for submitting code & software for further information.

## Data

Policy information about availability of data

All manuscripts must include a data availability statement. This statement should provide the following information, where applicable:

- Accession codes, unique identifiers, or web links for publicly available datasets
- A list of figures that have associated raw data
- A description of any restrictions on data availability

The data and primary codes that support the findings of this study are deposited in figshare (10.6084/m9.figshare.24720501).

# Field-specific reporting

Please select the one below that is the best fit for your research. If you are not sure, read the appropriate sections before making your selection.

☒ Life sciences ☐ Behavioural & social sciences ☐ Ecological, evolutionary & environmental sciences

For a reference copy of the document with all sections, see nature.com/documents/nr-reporting-summary-flat.pdf

# Life sciences study design

All studies must disclose on these points even when the disclosure is negative.

| | |
|---|---|
| Sample size | A total of 5 participants underwent single-neuronal recordings. Our main neuronal analysis is based on data from 272 neurons. The sample size is large enough for the statistical analyses we have performed in our study consistent with previously published data. |
| Data exclusions | No subjects were excluded from analysis. For neuronal analysis, prospective units that did not demonstrate waveform stability over the course of the experiment were excluded from the analysis based on standard criteria for off-line single unit sorting. Units that displayed overlap with neighboring across channels were excluded as well. |
| Replication | Similar results were observed across study participants for neuronal analyses. Other core analyses were performed on the population level, across individuals, in which case the variation in subject responses was incorporated into statistical testing/modeling. This variability in response is also shown through the plotting of individual raw data or data ranges in the figures as applicable. |
| Randomization | There was no randomization procedure for subject selection/enrollment since all participants performed the same task. Within a given experimental session, trial stimuli order was randomly generated to avoid effects potentially attributable to trial order. |
| Blinding | Blinding of analysis was not relevant since all subjects underwent similar task design. Blinding to clinical condition was performed. |

# Reporting for specific materials, systems and methods

We require information from authors about some types of materials, experimental systems and methods used in many studies. Here, indicate whether each material, system or method listed is relevant to your study. If you are not sure if a list item applies to your research, read the appropriate section before selecting a response.

### Materials & experimental systems

| n/a | Involved in the study | |
|---|---|---|
| ☒ | ☐ | Antibodies |
| ☒ | ☐ | Eukaryotic cell lines |
| ☒ | ☐ | Palaeontology and archaeology |
| ☒ | ☐ | Animals and other organisms |
| ☐ | ☒ | Human research participants |
| ☒ | ☐ | Clinical data |
| ☒ | ☐ | Dual use research of concern |

### Methods

| n/a | Involved in the study | |
|---|---|---|
| ☒ | ☐ | ChIP-seq |
| ☒ | ☐ | Flow cytometry |
| ☒ | ☐ | MRI-based neuroimaging |

## Human research participants

Policy information about studies involving human research participants

| | |
|---|---|
| Population characteristics | The participants were recruited for the study independently of underlying neuropathology, age or sex. The participants were drawn from the same population undergoing planned intraoperative neurophysiology for deep brain stimulator placement. |
| Recruitment | For neuronal recordings, participants underwent intraoperative neurophysiology as part of their planned deep brain stimulator (DBS) placement. Prior to consideration, candidates for the study were evaluated by a multidisciplinary team of neurologists, neurosurgeons, and neuropsychologists and decisions for surgery were unrelated to study participation. Once |



April 2020

and only after a patient was consented and scheduled for surgery, their candidacy for participation in the study was reviewed with respect to the following inclusion criteria: 18 years of age or older, right-hand dominant, capable to providing informed consent to study participation and intact language function with demonstration of English fluency by preoperative testing. All patients meeting inclusion criteria for intraoperative recordings were approached regarding study enrollment solely based on these criteria and not based on other features (e.g. study team's anticipated likelihood of patient choosing to enroll) in order to prevent selection bias as best possible. Only individuals who had planned awake microelectrode recording for target mapping were included.

After surgical consent was obtained and the participants were scheduled for surgery, an independent member of the research team approached the patient for study participation. They then filled a separate research consent if they wished to participate in the study. At any point in the study, including during the intraoperative phase, patients were freely able to withdraw from the study without any consequence to their clinical care.

| | |
|---|---|
| Ethics oversight | Massachusetts General Hospital Institutional Review Board |

Note that full information on the approval of the study protocol must also be provided in the manuscript.
